# Improving Screening Cut-Off Scores for DSM-5 Adolescent Anxiety Disorder Symptom Dimensions with the Screen for Child Anxiety Related Emotional Disorders

**DOI:** 10.1155/2014/517527

**Published:** 2014-01-29

**Authors:** William W. Hale III, Quinten A. W. Raaijmakers, Anne van Hoof, Wim H. J. Meeus

**Affiliations:** ^1^Research Center Adolescent Development, Utrecht University, P.O. Box 80.140, 3508 TC Utrecht, The Netherlands; ^2^Department of Development Psychology, Utrecht University, P.O. Box 80.140, 3508 TC Utrecht, The Netherlands; ^3^Department of Development Psychology, Tilburg University, P.O. Box 90.153, 5000 LE Tilburg, The Netherlands

## Abstract

Presently most adolescent anxiety disorder screening instruments make their determination of running a high risk for an anxiety disorder on the basis of a cut-off score measured by a single screening which can lead to false positives. Therefore, the goal of this study is to examine whether a repeated administration of the SCARED screening instrument for DSM-5 anxiety disorder symptoms could help in the detection of true positives while also avoiding false positives. Participants were 923 early adolescents from the general community. The adolescents' ages at the first annual screening ranged from 10 to 15 with an average of 12.5 years. In a prospective five-year longitudinal design, the adolescents completed the SCARED screening instrument for anxiety disorder symptoms on a yearly basis. To detect true positives and avoid false positives, the data were analyzed with Receiver Operating Characteristics (ROC) cut-off score analyses. ROC cut-off score analyses revealed that the sensitivity and specificity of high risk were greatly improved for repeated screenings above those of a single screening. The findings of this study demonstrate that a screening instrument (such as the SCARED) should be administered not just once but several times in order to better determine true positives and avoid false positives.

## 1. Introduction

Detection of anxiety disorder symptoms in adolescents from the general community can be conducted in two manners: either by a screening instrument (many times in the form of a questionnaire) or by a structured diagnostic interview. In respect to the latter, this is not only time consuming but also requires a trained interviewer to conduct the interview. On the other hand, screening instruments, like the Screen for Child Anxiety Related Emotional Disorders (SCARED), are relatively easy to complete, can be conducted in groups, and, in comparison to interviews, are quite inexpensive. It is for these reasons that screening instruments for anxiety disorder symptom dimensions, such as those described in the DSM-5 [1], have been widely employed both in the clinical and research fields.

In order to determine whether an individual score on a screening instrument for DSM disorders is within a clinical range, cut-off scores for these instruments have been devised. In previous screening instrument studies a single percentage of top scorers (i.e., prevalence rates of a mental disorder) were used in calculating high risk for the development of a mental disorder sometimes separately for males and females. This approach received criticism since such a procedure does not take potential false negatives (i.e., adolescents who have the disorder but are not detected) or false positives (i.e., adolescents who are supposedly detected but, in fact, do not have the disorder) into account [2]. Therefore, most recent studies of screening instrument cut-off scores employ Receiver Operating Characteristics (ROC) cut-off score analyses. As opposed to using a single percentage of top scorers to determine the clinical cut-off score, ROC cut-off score analysis is based on two separate indices: one for sensitivity which represents how accurate the instruments is able to select adolescents who actually have the disorder, and another for specificity which represents how accurate the instrument can specify those adolescents who do not have the disorder. These two indices are used to determine what the optimal balance is for both sensitivity and specificity [2, 3].

Furthermore a recent study [4] has suggested that in addition to ROC cut-off score analyses the problem of false positives can be further avoided by administrating the screening instrument at least twice to discriminate false positives from true positives. However, in our examination of the literature of screening instruments that have used ROC cut-off score analyses, administrating a screening instrument with repeated measurements has almost never been done. This lack of study on this topic is quite peculiar since the very reason for using a screening instrument in the psychiatric clinical practice is to detect if an adolescent runs a high risk for developing a DSM-5 disorder (in other words, that the adolescent is a true positive) and only then to conduct a structured psychiatric interview to determine if the adolescent not only runs a high risk for the disorder but also, in fact, has the disorder. If a screening instrument simply allows a large number of false positives to slip-through then not only is this financially counterproductive, but also it can needlessly frighten the adolescent. Again, the present-day approach to screening for psychiatric disorders is a strange state of affairs since large false positive findings for physiological screenings are lambasted both in the medical community and in the media, yet large false positive findings for psychiatric screenings are simply tolerated.

Hence, the goal of this study is to examine whether a yearly repeated administration of the SCARED (a well-known and validated screening instrument for DSM-5 anxiety disorder symptom dimensions) to adolescents from the general community could help in the detection of true positives and avoid false positives.

## 2. Methods

### 2.1. Participants

Data for this study were collected from adolescents as part of a prospective, longitudinal research project entitled CONAMORE (CONflict And Management Of RElationships), with a one-year interval between each of the annual screenings.

In the first annual screening of the study, 923 early adolescents participated. The student population was comprised of 468 (50.7%) males and 455 (49.3%) females. The age of the students ranged from 10 to 15 years (M_age_ = 12.4, SD = 0.59) at the first annual screening of this study. Sample attrition was 1.2% across the annual screenings.

Students filled in the Screen for Child Anxiety Related Emotional Disorders (SCARED) during the adolescents' homeroom study period at school. Before the study, the student and his/her parents received written information that described the research project. The written information asked if the student was willing to participate and, if so, to fill in the provided written informed consent form. Fewer than 1% elected not to participate. Consent was also obtained from all the participating schools. This study and its assent and consent documents were approved by the Research Review Board (Utrecht division) of the Dutch Institute for the Study of Education and Human Development (ISED).

### 2.2. Screening Instrument

The screening instrument for adolescent anxiety disorder symptom dimensions employed in this study was the original 38-item Screen for Child Anxiety Related Emotional Disorders [5, 6]. The SCARED is a self-report questionnaire that measures five anxiety disorder symptom dimensions in children and adolescents, namely, Generalized Anxiety Disorder (9 questionnaire items), Panic Disorder (13 questionnaire items), Separation Anxiety Disorder (8 questionnaire items), Social Anxiety Disorder (4 questionnaire items), and School Anxiety (or School Refusal; 4 questionnaire items). Apart from the School Anxiety symptom dimension, the other four symptom dimensions are mapped strongly onto DSM-5 anxiety disorder symptom dimensions. It should be noted that the SCARED is a screening instrument for anxiety disorder symptom dimensions and the scores of these dimensions do not reflect a clinical diagnosis.

In addition to the initial studies in clinical populations [5, 6], confirmatory factor analyses demonstrated that the SCARED possesses the same five-factor structure in adolescents from the general population as originally observed in clinically referred children and adolescents [7]. In a recent cross-cultural meta-analysis of its psychometric properties, the five-factor structure of the SCARED was borne out and demonstrated that the four SCARED scales related to the symptoms of the aforementioned DSM-5 anxiety disorders are valid and have robust psychometric properties [8].

Since the goal of this study is to examine whether a repeated administration of the SCARED screening instrument for DSM-5 anxiety disorder symptoms could help in the detection of true positives while at the same time avoid the identification of false positives, only the Generalized Anxiety Disorder, Panic Disorder, Separation Anxiety Disorder, and Social Anxiety Disorder scales of the SCARED were used in this study.

The adolescent rated each symptom item on a 3-point scale: 0 (almost never), 1 (sometimes), and 2 (often). The range of the scores for the various SCARED factors was: Generalized Anxiety Disorder (range: 0–18; sample item: “I worry about the future”), Panic Disorder (range: 0–26; sample item: “I am afraid without a reason”), Separation Anxiety Disorder (range: 0–16; sample item: “I am afraid to be alone”), and Social Anxiety Disorder (range: 0–8; sample item: “I am nervous around strangers”). The range of the internal consistency coefficients (Cronbach's alphas) of the SCARED factors for each annual screening of the study was, respectively, Generalized Anxiety Disorder .84–.87; Panic Disorder .83–.90; Separation Anxiety Disorder .68–.79; Social Anxiety Disorder .80–.86. Incidental missing values were estimated by Relative Mean Substitution [9] (Raaijmakers, 1999).

### 2.3. Data Analyses

To examine whether a longitudinal administration of the SCARED screening instrument for adolescents anxiety disorder symptom dimensions could help in the detection of true positives and avoid the identification of false positives, the data were analyzed with Latent Growth Modeling (LGM), Latent Class Growth Analysis (LCGM), and Receiver Operating Characteristics (ROC) analyses. Both LGM and LCGM analyses were needed to place the adolescents into high risk (the top 10% of those adolescents who scored the highest on each of the four anxiety disorder dimensions) and low-risk groups (the remaining 90% of the adolescent population) in order to determine the optimal cut-off score for each anxiety disorder symptom dimension. We choose the top 10% of adolescents who scored the highest since this is commonly used in screening instrument manuals (e.g., [10]) to determine adolescents at high risk. However the LGM and LCGM analyses, while important, are quite complex and are not central to understanding the finding of the research question. Hence the LGM and LCGM analyses will not receive further examination in this paper. The interested reader can obtain these analyses from the first author.

For the ROC cut-off score analyses, attention was first given to the Area Under the Curve (AUC) of each separate ROC analysis. As put by Murphy et al. [11], “The area under an ROC curve may be interpreted as an estimate of the probability that a randomly chosen ill person will…have a higher test score than a randomly chosen well person.” (page 552). In this study the AUC was used to examine if the screening of adolescents based on repeated screenings was better in detecting high risk adolescents than the screening based on the traditional single screening.

The sensitivity (i.e., percentage of false negatives) and specificity (i.e., percentage of false positives) for various cut-off scores for each of the subscales of the SCARED screening instrument were further examined by discriminating between false positives from true positives from the high risk group. This was also conducted to examine whether the screening of adolescents based on repeated screenings was better in detecting high risk adolescents than the traditional single screening.

## 3. Results

For each subscale of the SCARED, we performed three ROC cut-off score analyses to examine the sensitivity and specificity in detecting adolescents exhibiting a high risk anxiety trajectory.

The first ROC cut-off score analysis only used data from the first annual screening in order to provide a representation of how screening instruments are presently used in research and in the psychiatric clinical practice. The second ROC cut-off score analysis used data from the first and second annual screenings and the third ROC cut-off score analysis used data from the first through the third annual screenings in order to illustrate how repeated measurements increase screening accuracy above the traditional single screening procedure.

Table 1 reports the Area Under the Curve (AUC values) of each separate ROC cut-off score analysis. The use of repeated measurements significantly improved the performance of the screening of anxiety as demonstrated by the increasing AUC values and the significant differences between the AUC values, as evaluated with *z*-tests. While the screening based on three measurements was consistently the best in detecting high risk adolescents, the one exception was for the Panic Disorder symptoms dimension subscale in which screening twice was enough.

Table 2 reports the results of the second ROC analyses for cut-off scores. In addition to the sensitivity and specificity values, the absolute numbers of false negatives and false positives are also reported. The results indicate that a repeated measurements approach strongly increased the efficiency of the screening. Without deteriorating the accuracy in detecting high risk adolescents (i.e., sensitivity), the repeated measurement approach appeared to be far more effective in the avoidance of false positive cases (i.e., specificity).

## 4. Discussion

The findings of this study demonstrate that the yearly repeated administration of a screening instrument of adolescent anxiety disorder symptoms creates better cut-off scores and thereby enables a more accurate determination of the detection of true positives and also helps to avoid false positives. The findings of Tables 1 and 2 demonstrate that a procedure using repeating screenings was consistently better in detecting both high risk adolescents and avoiding false positives than the present-day cut-off score based on the single screening procedure. Additionally, the sensitivity and specificity values also confirmed that the cut-off scores of repeated screenings greatly decreased the number of false negative and false positives hence increasing the overall detection of true positives. For instance, if we use the cut-off score of Generalized Anxiety Disorder symptom dimension as an example (Table 2), it can be observed that the present-day one-time screening produces a specificity of 68%. This would mean that 289 adolescents would be incorrectly classified as running a high risk for Generalized Anxiety Disorder (in other words, these adolescents are false positives). However, if the screening is conducted twice, the specificity rises to 90% with only 90 adolescents being incorrectly classified as running a high risk for Generalized Anxiety Disorder (false positives). Finally, if the screening is conducted three times the specificity rises to 97% with only 26 false positives. So while repeated screenings will not totally eliminate the problem of incorrectly classifying adolescents as running a high risk for anxiety disorder symptomology (i.e., false positives), it greatly reduces its occurrences in comparison to the present-day approach of a one-time screening.

As was previously noted, screening instruments are relatively easy to complete and are quite inexpensive when compared to diagnostic interviews. Additionally, considering the relative ease of employing screening instruments, which can be filled in on line, it is quite conceivable that a repeated screening will provide more peace-of-mind to adolescents of not being unnecessary classified as high risk than is now the case. Furthermore, since a diagnostic interview, which is necessary before determining a diagnosis of a mental disorder, requires more time and expense than a screening instrument it would seem that repeated screenings are more cost efficient than a single screening.

With respect to the limitation of this study, it should be noted that the adolescents of this study came from the general population as opposed to adolescents diagnosed with a DSM-5 anxiety disorder. However, it has been noted in previous studies that community studies such as the present study are important because they circumvent the problem of referral bias that frequently occurs in the clinical setting [7] and that prospective, community studies may better characterize the course of mental disorders and thereby provide a better overall view of the general community where screening normally occurs [12].

Additionally, it should be stated that the time interval used in this study was one year between screenings. For the psychiatric clinical practice we would suggest that the clinician repeat the screening over a much short time interval, for example, over several weeks, since longer time intervals have a deteriorating effect on the predictive capacity of screening instruments such as the SCARED. However, the data from the prospective, longitudinal research project (CONAMORE), which was analyzed in this study, was collected on a yearly basis. Despite this limitation, the findings of this study would seem to indicate that replication studies of the SCARED that would employ even shorter intervals (such as several weeks) would have even more robust findings.

In conclusion, the findings of this study demonstrate that the screening instrument should be administered not just once, but several times in order to better determine true positives and avoid false positives.

## Figures and Tables

**Table 1:** Summary statistics of ROC analyses.

Scale	AUC	SE of AUC	*z* ^1^
Generalized Anxiety Disorder			
S1	.911	.030	
S1 + S2	.961	.011	2.31*
S1 + S2 + S3	.985	.008	2.50**
Panic Disorder			
S1	.833	.037	
S1 + S2	.890	.028	1.74*
S1 + S2 + S3	.916	.022	0.73
Separation Anxiety Disorder			
S1	.878	.031	
S1 + S2	.948	.011	3.01**
S1 + S2 + S3	.981	.004	3.99***
Social Anxiety Disorder			
S1	.903	.038	
S1 + S2	.970	.011	2.40**
S1 + S2 + S3	.992	.003	2.73**

*Note*. ^1^Test of the difference with previous screening (*z*-test value). AUC: Area Under the Curve, SE of AUC: standard error of Area Under the Curve, S1–S3: first through the third annual screenings.

*P < .05.  **P < .01.  ***P < .001.

**Table 2:** Cut-off scores, sensitivity (number of false negatives), and specificity (number of false positives) of the SCARED subscales in detecting adolescents with a high-risk level anxiety trajectory.

Cut-off score	Sensitivity^1^	Specificity^2^	Cut-off score	Sensitivity^1^	Specificity^2^
Generalized Anxiety Disorder			Panic Disorder		
Based on score at S1			Based on score at S1		
3.96	.90 (2)	.68 (289)	1.95	.86 (6)	.51 (430)
Based on scores at S1 + S2			Based on scores at S1 + S2		
23.04	.90 (2)	.90 (90)	20.02	.91 (4)	.70 (261)
Based on scores at S1 + S2 + S3			Based on scores at S1 + S2 + S3		
44.91	.90 (2)	.97 (26)	43.94	.86 (6)	.88 (108)

Separation Anxiety Disorder			Social Anxiety Disorder		
Based on score at S1			Based on score at S1		
4.00	.86 (6)	.83 (153)	3.00	.93 (2)	.68 (282)
Based on scores at S1 + S2			Based on scores at S1 + S2		
18.00	.93 (3)	.89 (99)	12.00	.93 (2)	.87 (116)
Based on scores at S1 + S2 + S3			Based on scores at S1 + S2 + S3		
30.96	.98 (1)	.92 (68)	22.00	.97 (1)	.96 (33)

*Note*. ^1^Sensitivity percentages; numbers between round brackets are the absolute number of false negatives. ^2^Specificity percentages; numbers between round brackets are the absolute number of false positives. S1–S3: First through the third annual screenings.
